# A Driving Method for Reducing Oil Film Splitting in Electrowetting Displays

**DOI:** 10.3390/membranes11120920

**Published:** 2021-11-24

**Authors:** Wenjun Zeng, Zichuan Yi, Yiming Zhao, Li Wang, Jitao Zhang, Xichen Zhou, Liming Liu, Feng Chi, Jianjun Yang, Chongfu Zhang

**Affiliations:** 1 College of Electron and Information, University of Electronic Science and Technology of China Zhongshan Institute, Zhongshan 528402, China; zwjcareer@163.com (W.Z.); ym1679@sina.com (Y.Z.); zxc_2021_4_15@163.com (X.Z.); liulmxps@126.com (L.L.); chif-eng@semi.ac.cn (F.C.); sdyman@uestc.edu.cn (J.Y.); cfzhang@uestc.edu.cn (C.Z.); 2 South China Academy of Advanced Optoelectronics, South China Normal University, Guangzhou 510006, China; 3 School of Information Engineering, Zhongshan Polytechnic, Zhongshan 528400, China; creekxi@163.com; 4 School of Mechanical and Electrical Engineering, Zhongshan Polytechnic, Zhongshan 528400, China; zhangjt4684@sina.com

**Keywords:** electrowetting displays (EWDs), driving waveform, oil film splitting, aperture ratio, quadratic function waveform

## Abstract

Electrowetting displays (EWDs) are one of the most potential electronic papers. However, they have the problem of oil film splitting, which could lead to a low aperture ratio of EWDs. In this paper, a driving waveform was proposed to reduce oil film splitting. The driving waveform was composed of a rising stage and a driving stage. First, the rupture voltage of oil film was analyzed by testing the voltage characteristic curve of EWDs. Then, a quadratic function waveform with an initial voltage was applied at the rising stage to suppress oil film splitting. Finally, a square wave was applied at the driving stage to maintain the aperture ratio of EWDs. The experimental results show that the luminance was increased by 8.78% and the aperture ratio was increased by 4.47% compared with an exponential function driving waveform.

## 1. Introduction

Electronic paper is a new type of reflective display device [1,2], which incorporates electrowetting displays (EWDs) [3,4] and electrophoretic displays (EPDs) [5,6]. These have the advantages of low power consumption, flexibility, readability in sunlight, and a wide viewing angle [7,8]. The power consumption of EPDs is lower than that of EWDs due to their bistable state, but their response speeds are slow, making it difficult to realize video playback [9]. On the contrary, EWDs effectively compensate for the limitations of EPDs in the two major performance areas of color display and video playback [10]. However, there are still design defects in EWDs which limit development, such as a low aperture ratio caused by oil film splitting [11]. Driving waveforms are a voltage sequence applied to EWDs, which can control the movement of oil films [12]. Therefore, the ability to reduce the oil film splitting in EWDs by optimizing driving waveforms is a significant one.

The aperture ratio of EWDs can reflect the degree of oil film contraction, which is related to driving voltages [13], pixel structure [14,15], and pixel materials [16]. Among them, the driving voltage plays a key role in the oil film contraction, and the value of the driving voltage depends on the design of the driving waveform. A driving waveform can be divided into a rising stage and a driving stage [17]. The design of the rising stage plays an important role in the suppression of oil film splitting. However, the rising stage was not designed in traditional driving waveforms, such as a pulse width modulation (PWM) driving waveform [18], which would cause serious oil film splitting in EWDs. An exponential function driving waveform was proposed to suppress the oil film splitting [19], because it can form a stable grayscale display by using this method. However, its initial voltage during the rising stage was lower than the rupture voltage of oil film, which prolonged the response time of EWDs. In order to solve this problem, an optimized voltage slope waveform was proposed, and additionally, a threshold voltage was added to the rising stage [20], so that the response time of EWDs could be effectively shortened. Furthermore, the driving stage was used to maintain the aperture ratio of EWDs, since the design of the driving stage plays an important role in achieving a high aperture ratio of EWDs. An amplitude–frequency mixed modulation driving method was proposed as the driving stage [21]; a high voltage was applied to achieve a target luminance, and the oil film was maintained by using a low voltage. However, the change of voltage amplitude could cause oil film oscillation, and the oil film could consequently be split [22]. So, the oil film oscillation was a problem which needed to be considered in the driving stage. To solve the oil film oscillation, an optimized alternating current (AC) driving waveform was proposed [23]. The characteristics of oil film stability were analyzed, and a stable aperture ratio could be achieved by using this method. The power consumption of EWDs was also a focus of the driving stage design; a sawtooth wave was proposed as the driving stage for ultra-low power consumption [24]. In addition, the model and theory of EWDs could provide a theoretical guidance for the design of driving waveforms, such as a dynamic electrowetting model [25,26], an oil film dynamic contraction model [27], charge trapping theory [28], and an oil film rupture model [29].

In this paper, a driving waveform, which was based on the principle of EWDs and oil film splitting theory, was proposed to reduce the oil film splitting. The driving waveform was composed of a rising stage and a driving stage. The rising stage was designed by analyzing the rupture process of oil film, and the driving stage was designed by the theory of power consumption calculation.

## 2. Principles and Methods

### 2.1. Principle of EWDs

**Figure 1:**
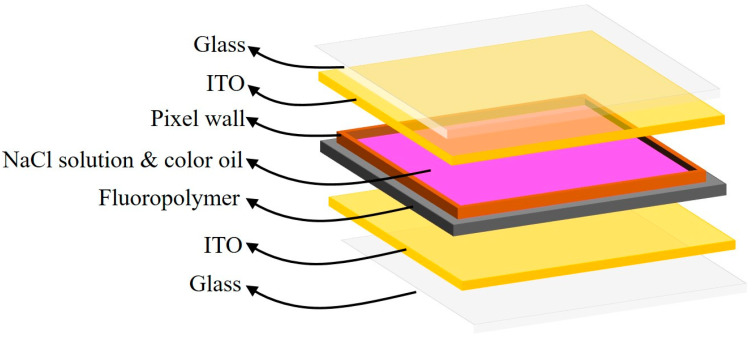
The structure of an EWD pixel. It is composed of a top glass, ITO electrodes, pixel walls, NaCl solution, color oil, a hydrophobic insulating layer (fluoropolymer), and a bottom glass. The color oil forms a thin film between the NaCl solution and the hydrophobic insulating layer, the pixel is in an “off” state.

Pixels of EWDs are fabricated using microtechnology. A pixel of EWDs is mainly composed of transparent glass, indium tin oxide (ITO) electrodes, pixel walls, conductive liquid (NaCl solution), color oil, and a hydrophobic insulating layer (fluoropolymer) [30,31,32], as shown in Figure 1. The color oil sticks to the hydrophobic insulating layer and forms a thin film when no voltage is applied. At this point, the pixel is in an “off” state, and it displays the color of the oil. Instead, the surface wettability of the hydrophobic insulating layer can be changed when a certain voltage is applied to the two electrodes. Then, the color oil contracts to a corner of the pixel. Now, the pixel is in an “on” state, and it displays the color of a substrate. Therefore, different grayscales can be displayed by controlling driving voltages [33]. In addition, the contact angle value of the oil film follows the Lippmann–Young equation, as shown in Equation (1) [34].

(1) $$cos\theta =1-\frac{CV^{2}}{2\gamma_{OW}}\;$$

where C is the capacitance of the pixel, V is a driving voltage applied to the pixel, and $\gamma_{OW}$ is the oil-water interfacial tension.

### 2.2. Oil Film Splitting

Oil film splitting describes a phenomenon in which the oil film splits into several pieces and cannot be reorganized during the contraction process [20]. The contraction process can be divided into an oil film rupture process and an oil film wetting process. The oil film rupture process refers to when the oil film is ruptured when the driving voltage reaches the rupture threshold voltage of oil film. The wetting process is when the oil film contracts to corners of the pixel, and its contact angle reaches to the Lippmann equilibrium contact angle [35]. The rupture threshold voltage, $V_{rp}$, of oil film can be obtained by Equation (2) [36].

(2) $$V_{rp}=\sqrt{\frac{\pi^{2}\gamma_{OW}{\left(h+\frac{\varepsilon_{oil}d}{\varepsilon_{FP}}\right)}^{3}}{\varepsilon_{0}\varepsilon_{oil}L^{2}}}$$

where h is the thickness of the oil film, $\varepsilon_{0}$ is the dielectric constant in vacuum, and $\varepsilon_{oil}$,$\;\varepsilon_{FP}$ are the dielectric constant of the oil film and the hydrophobic insulating layer, respectively. d is the thickness of the hydrophobic insulating layer, and L is the length of the pixel. It can be seen that the rupture threshold voltage is related to oil film thickness, dielectric layer thickness, and pixel size. The degree of oil film splitting mainly depends on the difference value between the driving voltage and the rupture threshold voltage of oil film. The oil film can maintain a relatively complete piece when the difference value is low. On the contrary, the oil film could be split into multiple small oil films when the difference value is high. Then, these small oil films could contract toward different corners in the wetting process, which would cause a low aperture ratio.

**Figure 2:**
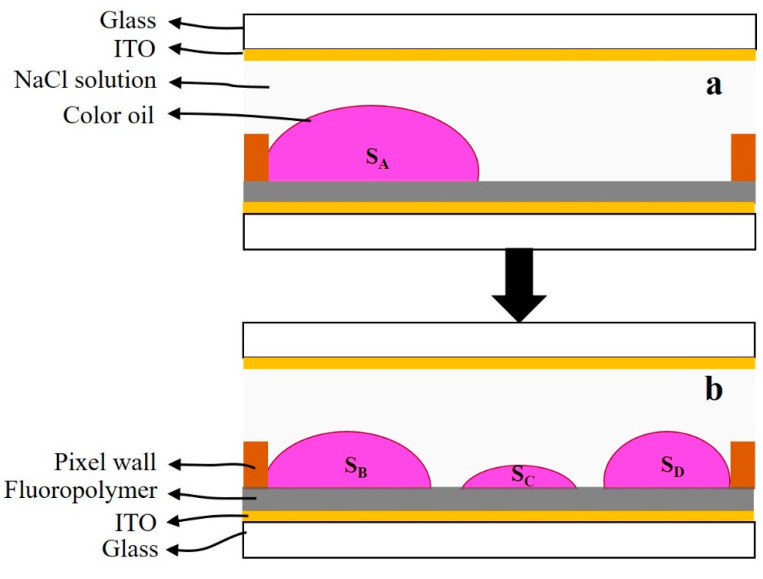
The situation of oil film contraction when a driving voltage is applied to a pixel. (**a**) A complete oil film contracts to a corner of the pixel without splitting; $S_{A}$ is the base area of the oil film. (**b**) The oil film was split into three pieces, and each piece contracts to different corners of the pixel; $S_{B}$, $S_{C}$ and $S_{D}$ are the base areas of the three split oil films.

The aperture ratio of EWDs is related to the base area occupied by the oil film in a single pixel. The value of aperture ratio, $A_{R}$, can be calculated by Equation (3) [37].

(3) $$A_{R}=1-\frac{S_{oil}}{S_{0}}=1-\frac{sin^{2}\theta {\left(1-\frac{3}{2}cos\theta_{0}+\frac{1}{2}cos^{3}\theta_{0}\right)}^{\frac{2}{3}}}{sin^{2}\theta_{0}{\left(1-\frac{3}{2}cos\theta +\frac{1}{2}cos^{3}\theta \right)}^{\frac{2}{3}}}$$

where $S_{oil}$ is a base area of oil film in the pixel, and $S_{0}$ is the area of the pixel. The base area of split oil films is larger than that of a complete oil film, as shown in Figure 2. $S_{A}$ is the base of the complete oil film; $S_{B}$, $S_{C}$ and $S_{D}$ are the base areas of split oil films. In the wetting process, the heights of the split oil films are lower than that of the complete oil film. Therefore, the relationship of their base area is $S_{A}<S_{B}+S_{C}+S_{D}$. It can be concluded that the value of $S_{oil}$ is increased when oil film splitting occurs, which decreases the aperture ratio of EWDs.

### 2.3. Design of Driving Waveform

**Figure 3:**
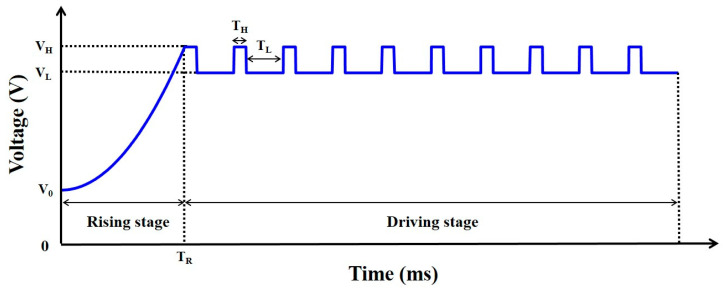
The proposed driving waveform for reducing oil film splitting. It is composed of a rising stage and a driving stage. The rising stage is a quadratic function waveform with an initial voltage; the driving stage is a square wave. $V_{0}$ is the initial voltage of the rising stage; $V_{L}$ is the low-level voltage of the driving stage. $V_{H}$ is the high-level voltage of the driving stage; $T_{R}$ is the rising time of the rising stage. $T_{H}$ is the driving time of the high level voltage, and $T_{L}$ is the driving time of the low level voltage.

We proposed a new driving waveform to reduce the oil film splitting. The driving waveform includes a rising stage and a driving stage, as shown in Figure 3. The design of the rising stage is a quadratic function waveform with an initial voltage, which can be expressed by Equation (4).

(4) $$U_{1}\left(t\right)=\frac{V_{H}-V_{0}}{T_{R}^{2}}t^{2}+V_{0}$$

where $V_{0}$ is the initial voltage of the rising stage, $V_{H}$ is the high-level voltage of the driving stage, $T_{R}$ is the rising time of the rising stage, and t is a time variable. The oil film remains in an equilibrium state when the driving voltage does not exceed the rupture threshold voltage of oil film. At this time, the aperture ratio of EWDs can remain unchanged, and the response time of EWDs is prolonged. Therefore, the initial voltage of the rising stage is set to solve this problem, which can improve the response speed of EWDs. Then, the driving voltage is raised from the initial voltage to the high-level voltage of the driving stage in a quadratic function waveform, because the quadratic function waveform can effectively prevent the serious oil film splitting caused by the excessive instantaneous electric field force. The design of the driving stage is a square wave to reduce power consumption of EWDs. The power consumption of the driving stage can be calculated by Equation (5).

(5) $$P=\frac{KV_{H}^{2}+\left(1-K\right)V_{L}^{2}}{R}$$

where K is the proportion of the high-level voltage, $V_{H}$, in a cycle of the square wave, $V_{L}$ is the low-level voltage of the driving stage, and R is the resistance of a single pixel. The driving stage of traditional driving waveforms is a direct current (DC) voltage, and its amplitude is assumed to $V_{F}$. Then, Equation (5) can be used to calculate the power consumption of traditional driving waveforms when $V_{L}=V_{F}$, K=0. The relationship between high and low-level voltages of the driving stage can be expressed by Equation (6).

(6) $$V_{H}+V_{L}=\frac{V_{F}}{2}$$

It can be deduced that the power consumption of the proposed driving stage is lower than that of the traditional driving waveform when K=14. Therefore, the time ratio of the high level and low level in the driving stage can be set to $T_{H}:T_{L}=1:3$. At this time, the power consumption can be effectively reduced.

## 3. Experimental Results and Discussion

### 3.1. Experimental Platform

**Figure 4:**
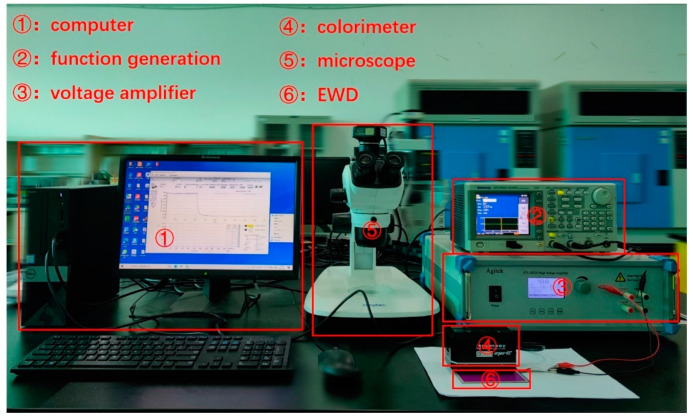
An experimental platform for testing the performance of the EWD. It was composed of a computer, a function generation, a voltage amplifier, a colorimeter, a microscope, and an EWD. The EWD was used as the tested object. The computer, the function generation, and the voltage amplifier were used to generate driving waveforms. Then, the colorimeter was used to obtain the luminance of the EWD, and the microscope was used to obtain the aperture ratio of the EWD.

We developed an experimental platform to verify the effectiveness of the driving waveform, as shown in Figure 4. The platform was composed of a driving system and a testing system. The driving system was composed of a computer (H430, Lenovo, Beijing, China), a function generator (AFG3022C, Tektronix, Beaverton, OR, USA), and a voltage amplifier (ATA-2022H, Agitek, Xian, China), which was used to generate driving waveforms. The testing system was composed of the computer, a colorimeter (Arges-45, Admesy, Ittervoort, The Netherlands), and a microscope (SZ680, Chongqing Optec Instrument Co., Ltd., Chongqing, China), which was used to record the luminance and aperture ratio data of EWDs.

In this experiment, an EWD was used as the tested object, and the parameters of the EWD are shown in Table 1. In the testing process, the driving waveform was edited by Arbexpress waveform editing software (V3.4, Tektronix, Beaverton, OR, USA) in the computer. Then, an edited driving waveform was imported into the function generator by a universal serial bus (USB) interface, and was then amplified by the voltage amplifier. Next, the EWD was driven by power from the voltage amplifier. The luminance and aperture ratio data of the EWD were collected by the colorimeter and the micrometer, respectively. Finally, the data were transmitted and recorded by the computer in real time.

### 3.2. Testing of the Rising Stage

**Figure 5:**
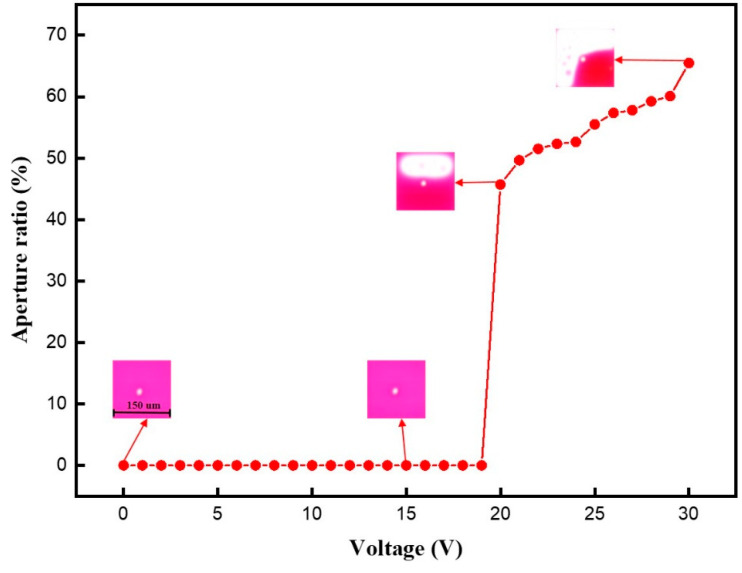
The aperture ratio was driven by different DC voltages. The aperture ratio was 0% when the voltage was 0–19 V. Then, the aperture ratio was 45.73% when the voltage was 20 V, and then it was increased with the increase in voltages. The maximum aperture ratio was 65.48%.

The aperture ratio of the EWD driven by DC voltages was tested to analyze the rupture threshold voltage of the oil film. The DC voltage was set to 0–30 V, and the experimental results are shown in Figure 5. When the DC voltage was 0–19 V, the aperture ratio remained unchanged, because the voltage had not reached the rupture threshold voltage of oil film at this time. Next, the aperture ratio began to rise when the driving voltage was 20 V, and then it was increased with the increase in the driving voltage. This phenomenon indicated that the oil film was ruptured when the driving voltage reached to 20 V, and that the oil film subsequently contracted to the corner of the pixel. Therefore, the rupture threshold voltage of the oil film was 20 V.

**Figure 6:**
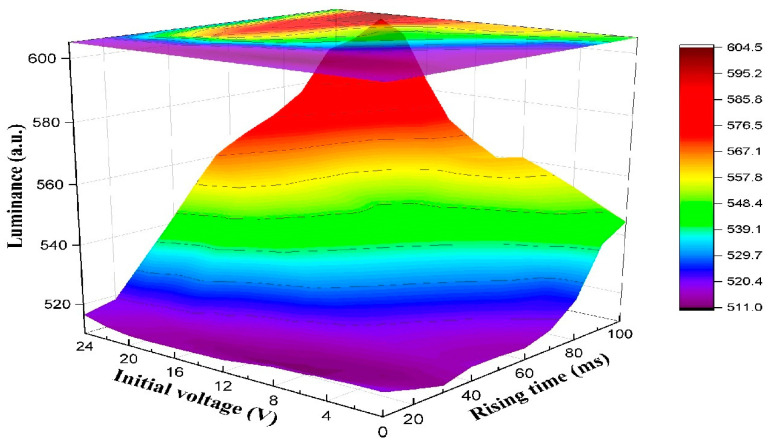
The luminance driven by different initial voltages and rising times of the rising stage in the proposed driving waveform. The luminance was below 520 when the rising time was 10–20 ms. When the rising time was 30–100 ms and the initial voltage was the same, the luminance could be increased with the increase in rising time. Additionally, the luminance was first increased with the increase in the initial voltage, but it had a downward trend when the initial voltage exceeded 20 V.

In the rising stage, the influence of the initial voltage and the rising time on the oil film splitting was analyzed. The initial voltage of the rising stage was set to 0–24 V, and the rising time of the rising stage was set to 10–100 ms. The luminance of different initial voltages and rising times are shown in Figure 6. It can be seen that the luminance was below 520 when the rising time was 10–20 ms. At this time, the intensity of the electric field applied to the EWD was increased rapidly due to the short rising time, which caused a serious oil film splitting. When the rising time was 30–100 ms, the luminance was first increased with the increase in the initial voltage, but it had a downward trend when the initial voltage exceeded 20 V. This phenomenon showed that the speed of oil film contraction could be increased with the increase in the applied electric field when the initial voltage was lower than the rupture threshold voltage. On the contrary, the increase in the initial voltage could lead to the increase in the oil film splitting degree when the initial voltage exceeded the rupture threshold voltage, which could decrease the luminance of the EWD. Therefore, the maximum luminance occurred when the initial voltage was 20 V. In addition, the luminance could be increased with the increase in rising time when the initial voltage was the same. It could be proven that the oil film can be effectively prevented from splitting by applying an electric field with a long rising time. However, the response speed of EWDs could be decreased when the rising time was long. Therefore, the rising time was set to 100 ms, which could effectively reduce the oil film splitting, and the response time of oil film cannot be excessively prolonged.

### 3.3. Testing of the Driving Stage

**Figure 7:**
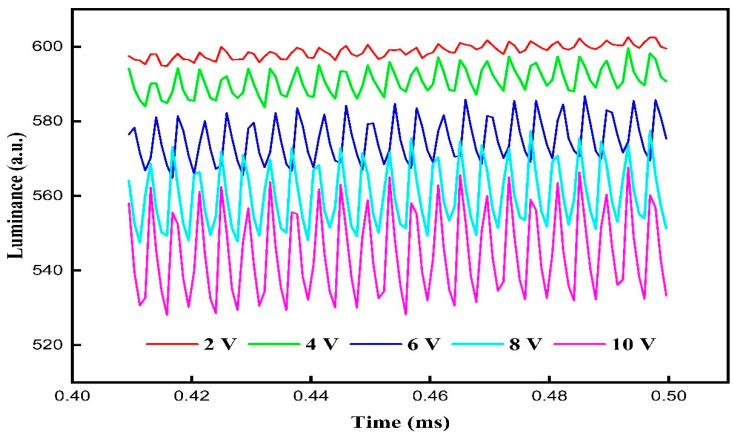
Luminance curves driven by different voltage amplitudes of the driving stage in the proposed driving waveform. The amplitude of luminance oscillation could be increased with the increase in voltage amplitudes. The minimum amplitude of luminance oscillation was 4.1 when the voltage amplitude was 2 V. In addition, the average luminance could be decreased with the increase in the voltage amplitude. The maximum average luminance was 598.79 when the voltage amplitude was 2 V.

In the driving stage, the influence of voltage amplitudes on the oil film oscillation was analyzed. $V_{F}$ was set to 30 V, which was same as traditional driving waveforms. Voltage amplitudes of the driving stage were set to 2, 4, 6, 8, and 10 V. Luminance curves driven by different voltage amplitudes are shown in Figure 7. It can be seen that the amplitude of luminance oscillations could be increased with the increase in the voltage amplitude. The minimum amplitude of the luminance oscillation was 4.1 when the voltage amplitude was 2 V, and the maximum amplitude of luminance oscillation was 25.29 when the voltage amplitude was 10 V. The change of driving voltage could cause oil film oscillation in this process. In addition, the increase in voltage amplitude could increase the degree of oil film oscillation, which could lead to the increase in luminance oscillation. At the same time, the average luminance could be decreased with the increase in the voltage amplitude. The maximum average luminance was 598.79 when the voltage amplitude was 2 V, and the minimum average luminance was 544.63 when the voltage amplitude was 10 V. This phenomenon showed that the splitting of oil film was greater when the voltage amplitude was increased. Therefore, the voltage amplitude of the driving stage was set to 2 V to stabilize the oil film.

### 3.4. Performance Comparison

**Figure 8:**
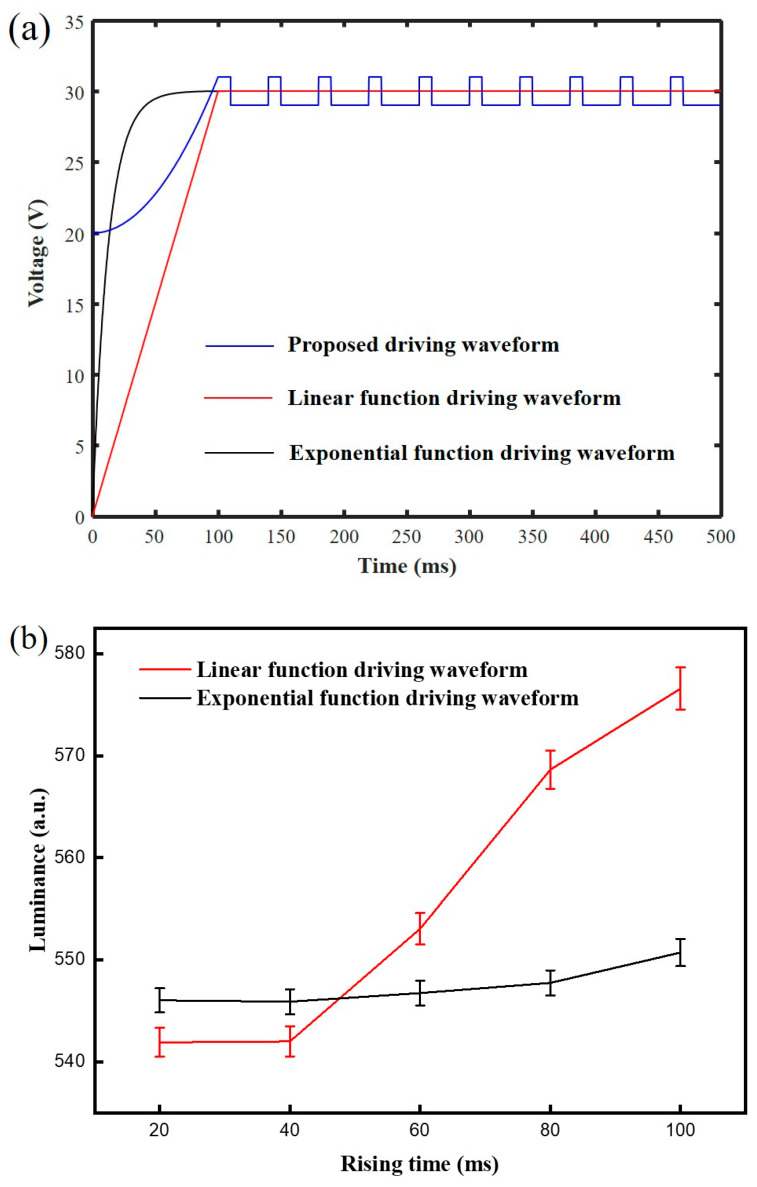

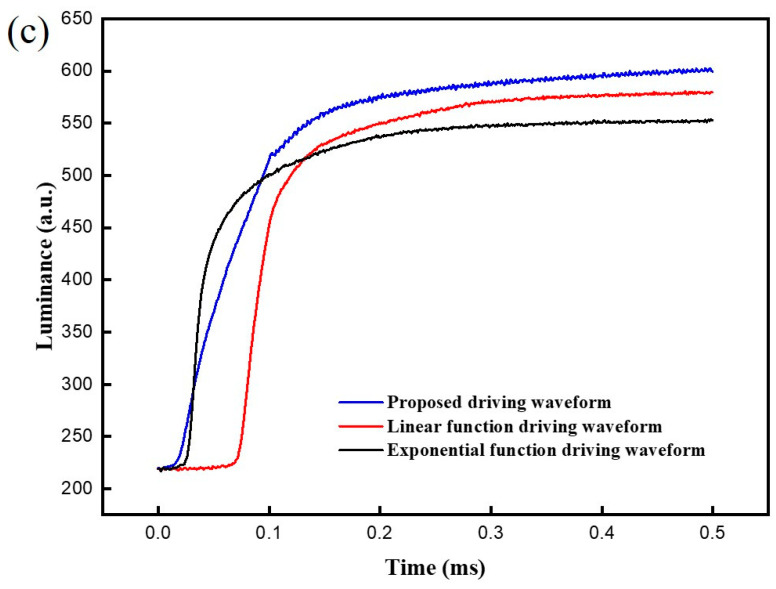
(**a**) Different driving waveforms for performance comparison. The blue line represents the proposed driving waveform; the red line the linear function driving waveform, and the black line the exponential function driving waveform. (**b**) The luminance driven by different rising times of the exponential function driving waveform and the linear function driving waveform. Luminance curves of the exponential function driving waveform and the linear function driving waveform were increased with the increase in rising time. (**c**) Luminance curves of different driving waveforms. The maximum luminance of the proposed driving waveform was 602.44, while the maximum luminance of the exponential function driving waveform and the linear function driving waveform were 579.97 and 553.81, respectively.

The exponential function driving waveform [19] and the linear function driving waveform [17] compared with the proposed driving waveform, as shown in Figure 8a. The luminance driven by different rising times of the exponential function driving waveform and the linear function driving waveform is shown in Figure 8b. It can be seen that the luminance curves of the exponential function driving waveform and the linear function driving waveform were positively correlated with the rising time, which had the same trend as the proposed driving waveform. The luminance can reach the maximum value at 100 ms. Therefore, rising times of the exponential function driving waveform and the linear function driving waveform were set to 100 ms, which was the same as the proposed driving waveform. Then, voltage amplitudes of the exponential function driving waveform and the linear function driving waveform were both set to 30 V. The initial voltage of the rising stage in the proposed driving waveform was set to 20 V; the high-level voltage and the low-level voltage in the driving stage were set to 31 V and 29 V, respectively. Luminance curves of different driving waveforms are shown in Figure 8c. It can be seen that the luminance of the proposed driving waveform increased the quickest at first, because its oil film ruptured speed was faster than that of other driving waveforms. Then, the oil film driven by the exponential function driving waveform was ruptured and its luminance was increased. The voltage rising rate of the exponential function driving waveform was higher than that of the proposed driving waveform, so the luminance increasing rate of the exponential function driving waveform can quickly exceed that of the proposed driving waveform. On the contrary, the rupture of oil film driven by the linear function driving waveform was the slowest, because it needed a long time to reach the rupture threshold voltage of oil film. When the luminance reached 500, the response time of the proposed driving waveform was 94.64 ms, while the exponential function driving waveform and the linear function driving waveform were 97.37 ms and 120.12 ms, respectively. When the luminance exceeded 500, the luminance of the exponential function driving waveform was increased slowly. On the contrary, the luminance of the proposed driving waveform and the linear function driving waveform exceeded that of the exponential function driving waveform. The luminance of the proposed driving waveform was higher than that of the linear function driving waveform. This phenomenon showed that the proposed driving waveform could effectively reduce the oil film splitting, while the exponential function driving waveform and the linear function driving waveform were affected by the oil film splitting, limiting the increase in luminance. Finally, the maximum luminance of the proposed driving waveform was 602.44, while the maximum luminance of the exponential function driving waveform and the linear function driving waveform were 579.97 and 553.81, respectively.

**Figure 9:**
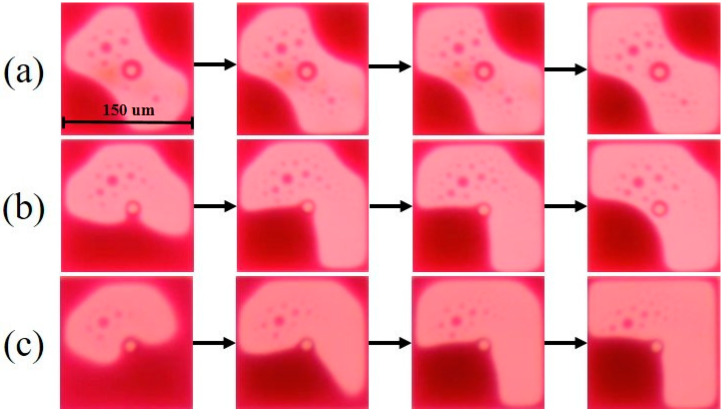
The driving process of different driving waveforms in a single EWD pixel. (**a**) Exponential function driving waveform. (**b**) Linear function driving waveform. (**c**) Proposed driving waveform. The splitting pieces of oil film driven by the proposed driving waveform was minimum; its aperture ratio was 66.8%. On the contrary, the splitting pieces of oil film driven by the exponential function driving waveform was maximum; its aperture ratio was 62.33%.

The driving process of different driving waveforms in a single EWD pixel was analyzed, as shown in Figure 9. It can be seen that the oil film driven by the exponential function driving waveform could split into five pieces in the rupture process. Then, the split oil film contracted to the corner of the pixel in the wetting process, but could not be recomposed into a complete oil film due to oil film splitting. The aperture ratio achieved by the exponential function driving waveform was 62.33%, and the oil film driven by the linear function driving waveform could split into three pieces in the rupture process, which was lower than that of the exponential function driving waveform. However, there was still an oil film which could not be recomposed. The aperture ratio achieved by the linear function driving waveform was 65.47%. On the contrary, the splitting of oil film driven by the proposed driving waveform was significantly reduced in the rupture process, and the oil film could recompose into a complete oil film in the wetting process. The aperture ratio achieved by the proposed driving waveform was 66.8%.

## 4. Conclusions

In this paper, a driving waveform was proposed to reduce oil film splitting in EWDs. The rising stage of the driving waveform was a quadratic function waveform, and the driving stage of the driving waveform was an optimized square wave. First, the rupture threshold voltage of oil film was obtained by testing the voltage characteristic curve of an EWD, so oil film splitting was effectively suppressed by optimizing parameters of the rising stage. Then, the average luminance of the EWD was increased, and the luminance oscillation of EWDs was decreased by setting an optimal voltage amplitude of the driving stage. Finally, the splitting pieces of oil film were decreased, and the aperture ratio of the EWD driven by the proposed driving waveform was increased and compared to the exponential function waveform and the linear function driving waveform. In summary, we designed a driving waveform for increasing the aperture ratio of EWDs and reducing oil film splitting, which provided a certain reference value for the field of EWDs.

## Figures and Tables

**Table 1:** Parameters of the EWD used in the experiment.

$\mathrm{Panel}\;\mathrm{Size}\;(cm^{2})$	Oil Color	Resolution	$\mathrm{Pixel}\;\mathrm{Size}\;(\mu m^{2})$	Pixel Wall Height (μm)	Hydrophobic Insulating Layer (μm)
10×10	Magenta	320×240	150×150	18	1

## Data Availability

Data are contained within the article.
